# On the minimum number of non-monochromatic simplices for Sperner labelings of a regular triangulation

**DOI:** 10.1371/journal.pone.0356507

**Published:** 2026-08-28

**Authors:** Luis Ángel Calvo Pascual, Susana Merchán Rubira, Dulcinea Raboso Paniagua, Javier Rodrigo Hitos, José Samuel Rodríguez García

**Affiliations:** 1 Comillas Pontifical University, Madrid, Spain; 2 Institute for Research in Technology (IIT), Madrid, Spain; 3 Polytechnic University of Madrid, Madrid, Spain; 4 CEU San Pablo University, Madrid, Spain; 5 Gran Capitán High School, Madrid, Spain; Minnan Normal University, CHINA

## Abstract

Motivated by an open problem in the literature stated by Mirzakhani and Vondrák, we give a lower bound of the number of non-monochromatic simplices for Sperner labelings of the vertices of a triangulation of a given *k*-simplex with vertices of integer coordinates. This triangulation maximizes the number of simplices over all the triangulations of the *k*-simplex with vertices of integer coordinates.

## 1. Introduction

This paper studies the minimum number of non-monochromatic simplices that can appear in a Sperner labeling of a triangulation of a simplex. Here, a simplex is called non-monochromatic if at least two of its vertices receive different labels. This question is related to hypergraph labeling problems and to several topics in combinatorial topology and discrete optimisation; for related work, see [1,2].

A main motivation comes from the hypergraph labeling problem, where one seeks a cut minimizing the sum of assignment costs of the vertices and the weights of the cut hyperedges. Lower bounds on the number of non-monochromatic simplices provide structural information for these problems; see [3–5] for background.

An open problem discussed in [6] is finding a lower bound for the number of non-monochromatic simplices in Sperner labelings of simplicial subdivisions (triangulations) of the simplex $\Delta_{k,q}$, where $\Delta_{k,q}$ denotes the set of vectors in ${\mathbb{R}}^{k}$ with non-negative entries that sum to *q*. Specifically, on the final page of [6], Mirzakhani and Vondrák proposed the following open question and conjecture:

*“For a Sperner-admissible labeling of a regular simplicial subdivision [...], what is the minimum possible number of non-monochromatic cells? [...]. We conjecture that for a fixed* j≤k *and as* q→∞*, the number of cells containing at least j colors is on the order of* $\Omega (q^{k-j})$*.”*

This leads to the regular triangulation setting considered in this paper. Let $\Delta_{k,q}=\{(x_{1},...,x_{k})\in {\mathbb{R}}_{\geq 0}^{k}|x_{1}+\cdots +x_{k}=q\}.$ For a Sperner labeling of the vertices of a triangulation of $\Delta_{k,q}$, let $m_{k,q}$ denote the minimum possible number of non-monochromatic simplices in a triangulation of $\Delta_{k,q}$. On the final page of [7], Mirzakhani and Vondrák conjectured that, for fixed j≤k and as q→∞, the number of cells containing at least *j* colors is of order $\Omega (q^{k-j})$. In the case *j* = 2, this predicts growth of order $\Omega (q^{k-2})$ for the quantity studied here.

In this paper, we study this question for the regular triangulation 𝒯, a symmetric triangulation of $\Delta_{k,q}$ whose vertices are the lattice points of the simplex. Our approach is to characterise 𝒯 in graph-theoretic terms and then relate it to the simplex-lattice hypergraph considered in [7]. This allows us to prove a lower bound and an upper bound of the same order for fixed *k*, and therefore to determine the order of growth of $m_{k,q}$ up to the multiplicative constant. In particular, this order of growth is $q^{k-2}$, which matches the asymptotic lower bound of the conjecture of [6] for *j* = 2. We also show that the bounds are exact in the initial cases *q* = 1 or *k* = 2, and we obtain the exact value of $m_{k,2}$.

We establish the following main results:

**Theorem 1.1** *The minimum number of non-monochromatic simplices for a Sperner labeling of the vertices of the regular triangulation* 𝒯 *of* $\Delta_{k,q}$ *satisfies*


mk,q≥(q+k−3k−2). (1.1)

This bound is proved to be tight for the initial cases *q* = 1 or *k* = 2. For *q* = 2 we give a better lower bound with an improvement of the multiplicative constant.

Note that this lower bound is on the order of $q^{k-2}$ as desired. Next, we give an upper bound for the minimum number of non-monochromatic simplices, which is $O(q^{k-2})$, with the multiplicative constant depending on the dimension *k*. Concretely,

**Theorem 1.2** *The minimum number of non-monochromatic simplices for a Sperner labeling of the vertices of the regular triangulation* 𝒯 *of* $\Delta_{k,q}$ *satisfies*


$$\begin{array}{c} m_{k,q}\leq q^{k-1}-(q-1{)}^{k-1}. \end{array}$$ (1.2)

This paper is organised as follows. Section 2 introduces definitions and notation used throughout. In Section 3, we present and characterise the regular triangulation 𝒯. Section 4 establishes a lower bound on the minimum number of non-monochromatic simplices within this triangulation. In Section 5, we determine an upper bound. Finally, in Section 6, we give some conclusions and future directions of work.

## 2. Basic concepts and notation

In this section, we fix notation and recall some definitions.

### Notation

- $\Delta_{k}$ is a simplex of *k* vertices, k≥2.
- $Conv\{v^{1},...,v^{k}\}$ is the convex hull of the vertices $v^{1},...,v^{k}\;.$
- $x_{i}$ is the *i*-th coordinate of the vector $x\in {\mathbb{R}}^{k}.$

**Definition 2.1** For k,q∈ℕ, k≥2, we define the *k-simplex* $\Delta_{k,q}$ as


$$\begin{array}{c} \Delta_{k,q}=\left\{(x_{1},...,x_{k})\in {\mathbb{R}}^{k}\;|\;\sum_{i=1}^{k}x_{i}=q,\;x_{i}\geq 0,\text{ for all }i=1,...,k\right\} \end{array}$$ (2.1)

$$\begin{array}{c} =Conv\{(q,0,...,0),...,(0,...,0,q)\}. \end{array}$$

**Definition 2.2** A *simplicial subdivision (*or *triangulation)* $T_{r}$ of a *k-*simplex $\Delta_{k}$ is a finite collection of simplices (called *cells*) satisfying the following conditions:

- The union of all simplices in $T_{r}$ is exactly $\Delta_{k}$, i.e.,
  $$\begin{array}{c} \underset{\Delta \in T_{r}}{⋃}\Delta =\Delta_{k}. \end{array}$$ (2.2)
- For any pair of simplices $\Delta^{1},\Delta^{2}\in T_{r}$, the intersection $\Delta^{1}\cap \Delta^{2}$ is either empty or a common face of both simplices.

We give the definition of Sperner labeling as stated in [6]. Despite it is not as general as the classical one, it gives a good fit to our development.

**Definition 2.3** Let *V* denote the set of vertices of a triangulation of $\Delta_{k,q}$. A labeling c:V→{1,...,k} is called a *Sperner labeling* if, for every vertex $v=(v_{1},v_{2},...,v_{k})\in V$, the following condition holds


$$\begin{array}{c} v_{i}=0\;\Rightarrow \;c(v)\text{≠}i. \end{array}$$ (2.3)

Now, we define a particular case of Sperner labeling (see [7]).

**Definition 2.4** The *first choice labeling* is a Sperner labeling defined on the set of vertices *V* as follows: for each vertex $v=(v_{1},v_{2},...,v_{k})\in V$,


$$\begin{array}{c} c(v)=\min\left\{i\in \{1,...,k\}|v_{i}>0\right\}. \end{array}$$ (2.4)

## 3. The regular triangulation

We will employ the simplicial subdivision of $\Delta_{k,q}$ defined in [7] (see also [8,9]), which we refer to as the *regular triangulation* 𝒯. The purpose of this section is to obtain a concrete description of 𝒯 in terms of adjacency among lattice points. To do so, we proceed in three steps. First, we introduce an auxiliary region $R_{k,q}$ and a triangulation $T_{k}$ of this region. Next, we encode the simplices of $T_{k}$ through the graph $G_{k}^{\prime }$ and then replace it with the more explicit graph $G_{k}^{\prime \prime }$, proving that both descriptions determine the same family of simplices, namely


$$\begin{array}{c} T_{k}=T_{k}^{\prime }=T_{k}^{\prime \prime }. \end{array}$$

Finally, we transport this description from $R_{k,q}$ to the discrete simplex $\Delta_{k,q}$ by means of the map ϕ, obtaining the graph $G_{k}$ on $\Delta_{k,q}\cap {\mathbb{Z}}^{k}$. This leads to Corollary 3.15, which characterises the regular triangulation 𝒯 as the family of convex hulls of *k* pairwise adjacent vertices in $G_{k}$. Before proceeding, we introduce the following preliminary definitions.

**Definition 3.1** Let k,q∈ℕ with k≥2. The simplex $R_{k,q}$ is given by


$$\begin{array}{c} R_{k,q}=\left\{y\in {\mathbb{R}}^{k-1}\;|\;0\leq y_{1}\leq y_{2}\leq \cdots \leq y_{k-1}\leq q\right\}. \end{array}$$ (3.1)

We denote by $W_{k,q}$ the set of integer lattice points contained in $R_{k,q}$, that is,


$$\begin{array}{c} W_{k,q}=R_{k,q}\cap {\mathbb{Z}}^{k-1}. \end{array}$$

**Definition 3.2** Let $w=(w_{1},...,w_{k-1})\in W_{k,q}$. A permutation π:{1,...,k−1}→{1,...,k−1} is said to be *consistent with w* if


$$\begin{array}{c} w_{i}=w_{i+1}\;\Rightarrow \;\pi (i)<\pi (i+1). \end{array}$$ (3.2)

**Definition 3.3** Let π:{1,...,k−1}→{1,...,k−1} be a permutation consistent with $w\in W_{k,q}$. We define the simplex


$$\begin{array}{c} \sigma (w,\pi )=\left\{y\in {\mathbb{R}}^{k-1}\;|\;y_{i}\geq 0,\;0\leq (y-w{)}_{\pi (1)}\leq \cdots \leq (y-w{)}_{\pi (k-1)}\leq 1\right\}. \end{array}$$ (3.3)

**Definition 3.4** The *triangulation* $T_{k}$ of the region $R_{k,q}$ is the collection of all simplices σ(w,π) arising from pair*s* (w,π), where π is a permutation consistent with $w\in W_{k,q}$, i.e.,


$$\begin{array}{c} T_{k}=\left\{\sigma (w,\pi )\;|\;w\in W_{k,q},\;\pi \text{ consistent with }w\right\}. \end{array}$$ (3.4)

**Definition 3.5** The *regular triangulation* 𝒯 of the discrete simplex $\Delta_{k,q}$ is the collection of simplices


$$\begin{array}{c} 𝒯=\left\{\phi (\sigma (w,\pi ))\;|\;w\in W_{k,q},\;\pi \text{ consistent with }w\right\}, \end{array}$$ (3.5)

where $\phi :R_{k,q}\to \Delta_{k,q}$ is the mapping given by


$$\begin{array}{c} \phi (y_{1},...,y_{k-1})=\left(y_{1},\;y_{2}-y_{1},\;y_{3}-y_{2},\;...,\;y_{k-1}-y_{k-2},\;q-y_{k-1}\right). \end{array}$$ (3.6)

We begin with the auxiliary triangulation $T_{k}$ of $R_{k,q}$ and describe its simplices by means of an associated graph.

**Definition 3.6** The graph $G_{k}^{\prime }$ is the pair $\left(V_{k}^{\prime },E_{k}^{\prime }\right)$, where*:*

- The vertex set is $V_{k}^{\prime }=W_{k,q}$.
- The edge set $E_{k}^{\prime }$ consists of pairs $\{w^{1},w^{2}\}\subset W_{k,q}$ such that there exist (w,π) that satisfy $w^{1},w^{2}\in \sigma (w,\pi ).$

**Definition 3.7** We define the collection $T_{k}^{\prime }$ as the set of convex hulls of *k* vertices in $W_{k,q}$ that are pairwise adjacent in the previous graph, that is,


$$\begin{array}{c} T_{k}^{\prime }=\left\{Conv(v^{1},...,v^{k})\;|\;v^{i}\in W_{k,q},\;\text{and }\{v^{i},v^{j}\}\in E_{k}^{\prime }\text{ for all }1\leq i<j\leq k\right\}. \end{array}$$ (3.7)

We now establish the relationship between the sets $T_{k}$ and $T_{k}^{\prime }$ defined in (3.4), (3.7), respectively.

**Proposition 3.8** *It is satisfied that* $T_{k}=T_{k}^{\prime }$*.*

*Proof.* Let $\sigma (w,\pi )\in T_{k}$ be a simplex. By construction, its *k* vertices $v^{1},...,v^{k}$ belong to $W_{k,q}$ and lie within σ(w,π), therefore $v^{1},...,v^{k}$ are pairwise adjacent in $G^{\prime }$ and then


$$\begin{array}{c} Conv(v^{1},...,v^{k})=\sigma (w,\pi )\in T_{k}^{\prime }, \end{array}$$

which shows that $T_{k}\subset T_{k}^{\prime }$.

Conversely, suppose there exist *k* pairwise adjacent vertices $v^{1},...,v^{k}$ such that


$$\begin{array}{c} Conv(v^{1},...,v^{k})\notin T_{k}. \end{array}$$

Since the union of all simplices in $T_{k}$ covers $R_{k,q}$, i.e.,


$$\begin{array}{c} R_{k,q}=\underset{\left(w,\pi \right)}{⋃}\sigma (w,\pi ), \end{array}$$ (3.8)

an edge of the convex hull $Conv(v^{1},...,v^{k})$ must intersect the interior of some simplex $\sigma (w,\pi )\in T_{k}$. Since this edge is an intersection of simplices of $T_{k}$, then $\sigma (w^{\prime },\pi )$ contains an interior point of σ(w,π) for some $w^{\prime }\in W_{k,q}$, a contradiction because the intersection of simplices of a triangulation is a common face. Therefore, our assumption must be false, and it follows that $T_{k}^{\prime }\subset T_{k}$ and then $T_{k}=T_{k}^{\prime }$ as desired. □

Proposition 3.8 shows that $T_{k}^{\prime }$ is a triangulation of $R_{k,q}$. We now introduce a more explicit graph-theoretic description of this triangulation.

**Definition 3.9** The graph $G_{k}^{\prime \prime }$ is the pair $(V_{k}^{\prime \prime },E_{k}^{\prime \prime }),$ where

- The vertex set is $V_{k}^{\prime \prime }=W_{k,q}$.
- For distinct vertices $w^{1},w^{2}\in W_{k,q}$, the edge $\left\{w^{1},w^{2}\right\}$ belongs to $E_{k}^{\prime \prime }$ if either
  $$\begin{array}{c} (w^{1}-w^{2}{)}_{i}\in \{0,1\},\;\text{for all }i=1,...,k-1, \end{array}$$ (3.9)
  or
  $$\begin{array}{c} (w^{1}-w^{2}{)}_{i}\in \{-1,0\},\;\text{for all }i=1,...,k-1. \end{array}$$ (3.10)

**Definition 3.10** The collection $T_{k}^{\prime \prime }$ is the set of convex hulls of *k* vertices in $W_{k,q}$ that are pairwise adjacent in the graph $G_{k}^{\prime \prime }$. That is,


$$\begin{array}{c} T_{k}^{\prime \prime }=\left\{Conv(v^{1},...,v^{k})\;|\;v^{i}\in W_{k,q},\;\{v^{i},v^{j}\}\in E_{k}^{\prime \prime },\text{ for all }1\leq i<j\leq k\right\}. \end{array}$$ (3.11)

**Proposition 3.11** *It is satisfied that* $G_{k}^{\prime }=G_{k}^{\prime \prime }$*.*

*Proof.* Recall that the graphs $G_{k}^{\prime }$ and $G_{k}^{\prime \prime }$ were introduced in Definition 3.6 and Definition 3.9, respectively. Since $V_{k}^{\prime }=V_{k}^{\prime \prime }=W_{k,q}$, it suffices to prove that $E_{k}^{\prime }=E_{k}^{\prime \prime }$. Assume that $\{w^{1},w^{2}\}\in E_{k}^{\prime }$, then $w^{1},w^{2}\in \sigma (w,\pi ),$ for some $w\in W_{k,q}$ and then


$$\begin{array}{c} 0\leq (w^{1}-w{)}_{\pi (1)}\leq ...\leq (w^{1}-w{)}_{\pi (k-1)}\leq 1. \end{array}$$

Since $(w^{1}-w{)}_{\pi (i)}\in \mathbb{Z},$ for all i=1,...,k−1, it follows that there exists an index *i* such that


$$\begin{array}{cc} (w^{1}-w{)}_{\pi (1)} & =...=(w^{1}-w{)}_{\pi (i)}=0,\\ (w^{1}-w{)}_{\pi (i+1)} & =...=(w^{1}-w{)}_{\pi (k-1)}=1. \end{array}$$ (3.12)

Similarly, for *w*^2^, there exists an index *j* such that


$$\begin{array}{cc} (w^{2}-w{)}_{\pi (1)} & =...=(w^{2}-w{)}_{\pi (j)}=0,\\ (w^{2}-w{)}_{\pi (j+1)} & =...=(w^{2}-w{)}_{\pi (k-1)}=1. \end{array}$$ (3.13)

Assume without loss of generality that *j* > *i*. Using (3.12) and (3.13), we obtain:

- for s≤i,
  $$\begin{array}{c} (w^{1}-w^{2}{)}_{\pi (s)}=(w^{1}-w{)}_{\pi (s)}-(w^{2}-w{)}_{\pi (s)}=0. \end{array}$$ (3.14)
- for i<s≤j,
  $$\begin{array}{c} (w^{1}-w^{2}{)}_{\pi (s)}=(w^{1}-w{)}_{\pi (s)}-(w^{2}-w{)}_{\pi (s)}=1-0=1. \end{array}$$ (3.15)
- for j<s≤k−1,
  $$\begin{array}{c} (w^{1}-w^{2}{)}_{\pi (s)}=(w^{1}-w{)}_{\pi (s)}-(w^{2}-w{)}_{\pi (s)}=1-1=0. \end{array}$$ (3.16)

Therefore, by (3.14), (3.15), and (3.16), we have $\{w^{1},w^{2}\}\in E_{k}^{\prime \prime }$. □

Now we show that $E_{k}^{\prime \prime }\subset E_{k}^{\prime }$ by induction on *k*. For *k* = 2, if $\{w^{1},w^{2}\}\in E_{2}^{\prime \prime }$, then we can assume without loss of generality that $w^{2}-w^{1}=1$. The permutation π(1)=1 is trivially admissible and satisfies


$$\begin{array}{c} 0\leq (w^{1}-w^{2}{)}_{\pi (1)}\leq 1, \end{array}$$

so $w^{1},w^{2}\in \sigma (w^{1},\pi )$ and then $\{w^{1},w^{2}\}\in E_{2}^{\prime }$.

Assume that $E_{k-1}^{\prime \prime }\subset E_{k-1}^{\prime }$ and consider $G_{k}^{\prime \prime }=(V_{k}^{\prime \prime },E_{k}^{\prime \prime })$ and $\{w^{1},w^{2}\}\in E_{k}^{\prime \prime }$. We can assume without loss of generality that $\left(w^{1}-w^{2}{)}_{i}\in \{0,1\right\}$ for i=1,...,k−1. We have two cases.

***Case 1.*** There exists an index *i* such that $(w^{1}{)}_{i}=(w^{1}{)}_{i+1}$ and $(w^{2}{)}_{i}=(w^{2}{)}_{i+1}$.

We consider


$$\begin{array}{c} w^{\prime 1}=(w_{1}^{1},...,w_{i}^{1},w_{i+2}^{1},...,w_{k-1}^{1}),\;w^{\prime 2}=(w_{1}^{2},...,w_{i}^{2},w_{i+2}^{2},...,w_{k-1}^{2})\in {\mathbb{Z}}^{k-2}\;. \end{array}$$

These satisfy that


$$\begin{array}{c} 0\leq (w^{\prime 1}{)}_{1}=(w^{1}{)}_{1}\leq (w^{\prime 1}{)}_{2}\leq ...\leq (w^{\prime 1}{)}_{k-2}\leq q, \end{array}$$

$$\begin{array}{c} 0\leq (w^{\prime 2}{)}_{1}=(w^{2}{)}_{1}\leq (w^{\prime 2}{)}_{2}\leq ...\leq (w^{\prime 2}{)}_{k-2}\leq q, \end{array}$$

so $w^{\prime 1},w^{\prime 2}\in R_{k-1,q}\cap {\mathbb{Z}}^{k-2}=W_{k-1,q}$.

Since $(w^{\prime 1}-w^{\prime 2}{)}_{i}$ corresponds to $(w^{1}-w^{2}{)}_{j}$, for some j∈{1,...,k−2}, and $\left(w^{1}-w^{2}{)}_{j}\in \{0,1\right\}$ by assumption, it follows that $\left(w^{\prime 1}-w^{\prime 2}{)}_{i}\in \{0,1\right\}$, for all i=1,...,k−2. Consequently, $\{w^{\prime 1},w^{\prime 2}\}\in E_{k-1}^{\prime \prime }$, and by the induction hypothesis, $\{w^{\prime 1},w^{\prime 2}\}\in E_{k-1}^{\prime }$. Therefore, there exists $w^{\prime }\in W_{k-1,q}$ and a permutation π such that $w^{\prime 1},w^{\prime 2}\in \sigma (w^{\prime },\pi )$. We also have that $E_{k-1}^{\prime \prime }=E_{k-1}^{\prime }$ and then $G_{k-1}^{\prime \prime }=G_{k-1}^{\prime }$, so $T_{k-1}^{\prime \prime }=T_{k-1}^{\prime }$ and the simplices of the triangulation of $R_{k-1,q}$ are the convex hulls of k−1 vertices pairwise adjacent in $G_{k-1}^{\prime \prime }$.

The map


$$\begin{array}{c} f(x_{1},...,x_{i},x_{i+1},...,x_{k-2})=(x_{1},...,x_{i},x_{i},x_{i+1},...,x_{k-2}) \end{array}$$

defines a bijection between $W_{k-1,q}$ and $W_{k,q}\cap \{(x_{1},...,x_{k-1})\;|\;x_{i}=x_{i+1}\}$, where the inverse is given by


$$\begin{array}{c} f^{-1}(x_{1},...,x_{i},x_{i},x_{i+1},...,x_{k-2})=(x_{1},...,x_{i},x_{i+1},...,x_{k-2}). \end{array}$$

Since *f* just duplicates a coordinate and *f*^-1^ removes the repeated coordinate, it follows that edges of $E_{k-1}^{\prime \prime }$ are mapped to edges of $E_{k}^{\prime \prime }$ whose vertices lie in


$$\begin{array}{c} W_{k,q}\cap \{(x_{1},...,x_{k-1})|x_{i}=x_{i+1}\}. \end{array}$$

Conversely, edges of $E_{k}^{\prime \prime }$ with vertices in $W_{k,q}\cap \{(x_{1},...,x_{k-1})|x_{i}=x_{i+1}\}$ are mapped back to edges of $E_{k-1}^{\prime \prime }$ by *f*^-1^. Therefore, $G_{k-1}^{\prime \prime }$ is isomorphic to the induced subgraph


$$\begin{array}{c} G_{k}^{\prime \prime }{|}_{W_{k,q}\cap \{(x_{1},...,x_{k-1})|x_{i}=x_{i+1}\}}. \end{array}$$

In this way, the set of convex hulls of k−1 vertices of $W_{k,q}\cap \{(x_{1},...,x_{k-1})|x_{i}=x_{i+1}\}$ pairwise adjacent in $G_{k}^{\prime \prime }$ is a triangulation of


$$\begin{array}{c} R_{k,q}\cap \{(x_{1},...,x_{k-1})|x_{i}=x_{i+1}\}, \end{array}$$

we call it *T*, and $w^{1},w^{2}$ share a simplex of *T*, since $w^{\prime 1},w^{\prime 2}$ share a simplex of $T_{k-1}^{\prime \prime }$.

Moreover, for each $\sigma (w,\pi )\in T_{k}^{\prime }$ containing k−1 vertices of


$$\begin{array}{c} W_{k,q}\cap \{(x_{1},...,x_{k-1})\in {\mathbb{Z}}^{k-1}|x_{i}=x_{i+1}\}, \end{array}$$

the intersection


$$\begin{array}{c} \sigma (w,\pi )\cap \{(x_{1},...,x_{k-1})\in {\mathbb{R}}^{k-1}|x_{i}=x_{i+1}\} \end{array}$$

is a simplex of


$$\begin{array}{c} R_{k,q}\cap \{(x_{1},...,x_{k-1})\in {\mathbb{R}}^{k-1}|x_{i}=x_{i+1}\}. \end{array}$$

Since these k−1 vertices are pairwise adjacent in $G_{k}^{\prime \prime }$, it follows that


$$\begin{array}{c} \sigma (w,\pi )\cap \{(x_{1},...,x_{k-1})\in {\mathbb{R}}^{k-1}|x_{i}=x_{i+1}\}\in T. \end{array}$$

We denote these simplices by $\sigma^{\prime }(w,\pi )$.

We have


$$\begin{array}{c} \underset{\left(w,\pi \right)}{⋃}\left(\sigma^{\prime }(w,\pi )\cap \{(x_{1},...,x_{k})|x_{i}=x_{i+1}\}\right)=R_{k,q}\cap \{(x_{1},...,x_{k})|x_{i}=x_{i+1}\}. \end{array}$$

Otherwise,


$$\begin{array}{c} R_{k,q}\cap \{(x_{1},...,x_{k})|x_{i}=x_{i+1}\}-\underset{\left(w,\pi \right)}{⋃}\left(\sigma^{\prime }(w,\pi )\cap \{(x_{1},...,x_{k})|x_{i}=x_{i+1}\}\right) \end{array}$$

is a nonempty open subset of $R_{k,q}\cap \{(x_{1},...,x_{k})|x_{i}=x_{i+1}\}$, so it has dimension k−2. But since


$$\begin{array}{c} \underset{\left(w,\pi \right)}{⋃}\left(\sigma (w,\pi )\cap \{(x_{1},...,x_{k})|x_{i}=x_{i+1}\}\right)=R_{k,q}\cap \{(x_{1},...,x_{k})|x_{i}=x_{i+1}\}, \end{array}$$

we would have that


$$\begin{array}{c} R_{k,q}\cap \{(x_{1},...,x_{k})|x_{i}=x_{i+1}\}-\underset{\left(w,\pi \right)}{⋃}\left(\sigma^{\prime }(w,\pi )\cap \{(x_{1},...,x_{k})|x_{i}=x_{i+1}\}\right) \end{array}$$

is the union of a finite number of sets with dimension <k−2, a contradiction.

We also have that


$$\begin{array}{c} \left(\sigma^{\prime }(u,\pi )\cap \{(x_{1},...,x_{k})|x_{i}=x_{i+1}\})\cap (\sigma^{\prime }(v,\pi )\cap \{(x_{1},...,x_{k})|x_{i}=x_{i+1}\}\right) \end{array}$$

is either empty or a common face for $u,v\in W_{k,q}$, u≠v, since $\sigma^{\prime }(u,\pi )\cap \sigma^{\prime }(v,\pi )$ is either empty or a common face for $u,v\in W_{k,q}$, u≠v.

It follows that


$$\begin{array}{c} \left\{\sigma^{\prime }(w,\pi )\cap \{(x_{1},...,x_{k})|x_{i}=x_{i+1}\}\right\} \end{array}$$

is a triangulation of


$$\begin{array}{c} R_{k,q}\cap \{(x_{1},...,x_{k})|x_{i}=x_{i+1}\} \end{array}$$

included in *T*, and then


$$\begin{array}{c} T=\left\{\sigma^{\prime }(w,\pi )\cap \{(x_{1},...,x_{k})|x_{i}=x_{i+1}\}\right\}. \end{array}$$

This implies that $w^{1},w^{2}\in \sigma^{\prime }(w,\pi )$, for some $w\in W_{k,q}$, and then $\{w^{1},w^{2}\}\in E_{k}^{\prime }$. Therefore, $E_{k}^{\prime \prime }\subset E_{k}^{\prime }$ in this case.

***Case 2.*** Suppose there does not exist an index *i* such that


$$\begin{array}{c} (w^{1}{)}_{i}=(w^{1}{)}_{i+1}\;\text{and}\;(w^{2}{)}_{i}=(w^{2}{)}_{i+1}. \end{array}$$

We can assume that $i_{1}<i_{2}<...<i_{j}$, $i_{j+1}<i_{j+2}<...<i_{k-1}$, for some *j*, and $(w^{1}-w^{2}{)}_{i_{s}}=0$ for 1≤s≤j, while $(w^{1}-w^{2}{)}_{i_{s}}=1$ for j<s≤k−1.

We define the following permutation of {1,...,k−1}: $\pi (t)=i_{t}$. Let us see that π(t) is consistent with *w*^2^ if either $(w^{2}{)}_{j}\text{≠}(w^{2}{)}_{j+1}$ or $(w^{2}{)}_{j}=(w^{2}{)}_{j+1}$ and $i_{j}<i_{j+1}$.

Indeed, π(t) is strictly increasing except for (maybe) *j*,*j* + 1, so if $(w^{2}{)}_{j}\text{≠}(w^{2}{)}_{j+1}$, then π(t) is consistent with *w*^2^. If $(w^{2}{)}_{j}=(w^{2}{)}_{j+1}$ and $i_{j}<i_{j+1}$ (that is to say, if $(w^{1}{)}_{i}=(w^{2}{)}_{i}$ for i≤j), then π(j)<π(j+1) and π(t) is strictly increasing, so π(t) is consistent with *w*^2^.

Moreover, we have


$$\begin{array}{c} (w^{1}-w^{2}{)}_{\pi (1)}=...=(w^{1}-w^{2}{)}_{\pi (j)}=0 \end{array}$$

and


$$\begin{array}{c} (w^{1}-w^{2}{)}_{\pi (j+1)}=...=(w^{1}-w^{2}{)}_{\pi (k-1)}=1. \end{array}$$

Hence, it follows that $w^{1}\in \sigma (w^{2},\pi )$ and then $\{w^{1},w^{2}\}\in E_{k}^{\prime }$ in this case.

If $(w^{2}{)}_{j}=(w^{2}{)}_{j+1}$ and $i_{j+1}<i_{j}$, then $(w^{1}{)}_{j}\text{≠}(w^{1}{)}_{j+1}$ by the assumption.

Since $\left(w^{1}-w^{2}{)}_{t}\in \{0,1\right\}$ for every *t*, the former implies that $(w^{1}{)}_{j}=(w^{2}{)}_{j}$ and $(w^{1}{)}_{j+1}=(w^{2}{)}_{j+1}+1$, so $i_{1}\leq j<j+1\leq i_{k-1}$, and *t*hen the permutation


$$\begin{array}{c} \begin{array}{cc} \pi (1) & =i_{2},...,\pi (j-1)=i_{j},\pi (j)=i_{1},\\ \pi (j+1) & =i_{k-1},\pi (j+2)=i_{j+1},...,\pi (k-1)=i_{k-2}. \end{array} \end{array}$$

satisfies that if $(w^{2}{)}_{i}=(w^{2}{)}_{i+1}$, then π(i)<π(i+1), except for the cases i=j−1, *i* = *j* + 1. So, if $(w^{2}{)}_{j-1}\text{≠}(w^{2}{)}_{j}$ and $(w^{2}{)}_{j+1}\text{≠}(w^{2}{)}_{j+2}$, then π is admissible.

In the case $(w^{2}{)}_{j+1}=(w^{2}{)}_{j+2}$, we obtain


$$\begin{array}{c} (w^{1}{)}_{j+2}\geq (w^{1}{)}_{j+1}=(w^{2}{)}_{j+1}+1=(w^{2}{)}_{j+2}+1. \end{array}$$

As a result, $(w^{1}{)}_{j+2}=(w^{2}{)}_{j+2}+1=(w^{2}{)}_{j+1}+1=(w^{1}{)}_{j+1}$, a contradiction.

Similarly, if $(w^{2}{)}_{j-1}=(w^{2}{)}_{j}$, then


$$\begin{array}{c} (w^{1}{)}_{j}=(w^{2}{)}_{j}=(w^{2}{)}_{j-1}\leq (w^{1}{)}_{j-1}, \end{array}$$

so $(w^{1}{)}_{j}=(w^{1}{)}_{j-1}$, a contradiction. Therefore, π is admissible, with


$$\begin{array}{c} (w^{1}-w^{2}{)}_{\pi (1)}=...=(w^{1}-w^{2}{)}_{\pi (j)}=0 \end{array}$$

and


$$\begin{array}{c} (w^{1}-w^{2}{)}_{\pi (j+1)}=...=(w^{1}-w^{2}{)}_{\pi (k-1)}=1. \end{array}$$

From which it follows that $w^{1}\in \sigma (w^{2},\pi )$ and then $\{w^{1},w^{2}\}\in E_{k}^{\prime }$, so $E_{k}^{\prime \prime }\subset E_{k}^{\prime }$ as desired. □

**Corollary 3.12** *The triangulations* $T_{k}$*,* $T_{k}^{\prime }$*, and* $T_{k}^{\prime \prime }$ *are equal.*

*Proof.* Recall that $T_{k}$, $T_{k}^{\prime }$, and $T_{k}^{\prime \prime }$ are defined by (3.4), (3.7), and (3.11), respectively. By Proposition 3.11, the graphs $G_{k}^{\prime }$ and $G_{k}^{\prime \prime }$ are isomorphic, then the sets of *k* vertices that are pairwise adjacent in $G_{k}^{\prime }$ and $G_{k}^{\prime \prime }$ coincide. Thus, the collections of simplices defined as the convex hulls of such sets are equal, that is, $T_{k}^{\prime }=T_{k}^{\prime \prime }$.

On the other hand, by Proposition 3.8, we have $T_{k}=T_{k}^{\prime }$.

Combining these equalities, it follows that


$$\begin{array}{c} T_{k}=T_{k}^{\prime }=T_{k}^{\prime \prime }. \end{array}$$

**Definition 3.13** The graph $G_{k}$ is the pair $\left(V_{k},E_{k}\right)$, where □

- The vertex set is $V_{k}=\Delta_{k,q}\cap {\mathbb{Z}}^{k}$.
- For distinct vertices $v^{1},v^{2}\in V_{k}$, the edge $\left\{v^{1},v^{2}\right\}$ belongs to $E_{k}$ if
  $$\begin{array}{c} (v^{1}-v^{2}{)}_{i}\in \{-1,0,1\}\;\text{for all }i=1,...,k, \end{array}$$ (3.17)
  and the number of entries equal to 1 and −1 are equal and when reading the nonzero entries from left to right, the signs alternate.

We now establish the relationship between the graphs $G_{k}^{\prime \prime }$ and $G_{k}$ introduced in Definitions 3.9 and 3.13, respectively.

**Proposition 3.14** $G_{k}^{\prime \prime }$ *is isomorphic to* $G_{k}$.

*Proof.* The mapping $\phi {|}_{W_{k,q}}:W_{k,q}\to \Delta_{k,q}\cap {\mathbb{Z}}^{k}$, where ϕ is given by (3.6), is bijective, with inverse


$$\begin{array}{c} \phi^{-1}(x_{1},...,x_{k})=(x_{1},x_{1}+x_{2},x_{1}+x_{2}+x_{3},...,x_{1}+x_{2}+...+x_{k-1}). \end{array}$$ (3.18)

If $\{w^{1},w^{2}\}\in E_{k}^{\prime \prime }$, we may assume without loss of generality that $\left(w^{1}-w^{2}{)}_{i}\in \{0,1\right\}$ for all i=1,...,k−1. Let *i*_0_ denote the smallest index such that $(w^{1}-w^{2}{)}_{i_{0}}=1$.

If *i*_0_ > 1, then for all *i* < *i*_0_, we have


$$\begin{array}{c} (\phi (w^{1}){)}_{i}=(w^{1}{)}_{i}-(w^{1}{)}_{i-1}=(w^{2}{)}_{i}-(w^{2}{)}_{i-1}=(\phi (w^{2}){)}_{i}, \end{array}$$ (3.19)

and


$$\begin{array}{c} (\phi (w^{1}){)}_{i_{0}}=(w^{1}{)}_{i_{0}}-(w^{1}{)}_{i_{0}-1}=(w^{2}{)}_{i_{0}}+1-(w^{2}{)}_{i_{0}-1}=(\phi (w^{2}){)}_{i_{0}}+1. \end{array}$$ (3.20)

If *i*_0_ = 1, the relation simplifies to


$$\begin{array}{c} (\phi (w^{1}){)}_{i_{0}}=(w^{1}{)}_{i_{0}}=(w^{2}{)}_{i_{0}}+1=(\phi (w^{2}){)}_{i_{0}}+1. \end{array}$$ (3.21)

Next, let *i*_1_ be the smallest index greater than *i*_0_ such that $(w^{1}-w^{2}{)}_{i_{1}}=0$, if such an index exists. If $i_{1}>i_{0}+1$, then for all $i_{0}<i<i_{1}$ we have


$$\begin{array}{c} (\phi (w^{1}){)}_{i}=(w^{1}{)}_{i}-(w^{1}{)}_{i-1}=(w^{2}{)}_{i}+1-\left((w^{2}{)}_{i-1}+1\right)=(\phi (w^{2}){)}_{i}, \end{array}$$ (3.22)

and at *i*_1_ we have


$$\begin{array}{c} (\phi (w^{1}){)}_{i_{1}}=(w^{1}{)}_{i_{1}}-(w^{1}{)}_{i_{1}-1}=(w^{2}{)}_{i_{1}}-\left((w^{2}{)}_{i_{1}-1}+1\right)=(\phi (w^{2}){)}_{i_{1}}-1. \end{array}$$ (3.23)

Using (3.19)–(3.23) (or (3.21) when *i*_0_ = 1), and iterating the same argument with *i*_2_, *i*_3_, etc, we conclude that the nonzero entries of $\phi (w^{1})-\phi (w^{2})$ alternate between 1 and −1. Moreover, we have the same number of 1’s and −1’s since


$$\begin{array}{c} (\phi (w^{1})-\phi (w^{2}){)}_{1}+...+(\phi (w^{1})-\phi (w^{2}){)}_{k}=q-q=0. \end{array}$$

Therefore, the numbers of 1’s and −1’s coincide, and hence $\{\phi (w^{1}),\phi (w^{2})\}\in E_{k}$.

Conversely, if $\{v^{1},v^{2}\}\in E_{k}$, then we can assume without loss of generality that the lowest index *i* such that $(v^{1}-v^{2}{)}_{i}\text{≠}0$, denoted by *i*_1_, satisfies $(v^{1}-v^{2}{)}_{i_{1}}=1$. If $i_{j}$, *j* = 1, ..., 2*t*, is the ordered list of indices such that $(v^{1}-v^{2}{)}_{i_{j}}\text{≠}0$, then we have $(v^{1}-v^{2}{)}_{i_{j}}=1$ for odd *j* and $(v^{1}-v^{2}{)}_{i_{j}}=-1$ for even *j*.

Consequently, if *i*_1_ > 1, then, for *i* < *i*_1_,


$$\begin{array}{c} (\phi^{-1}(v^{1}){)}_{i}=\sum_{j=1}^{i}(v^{1}{)}_{j}=\sum_{j=1}^{i}(v^{2}{)}_{j}=(\phi^{-1}(v^{2}){)}_{i}, \end{array}$$ (3.24)

where


$$\begin{array}{c} (\phi^{-1}(v^{1}){)}_{i_{1}}=\sum_{j=1}^{i_{1}}(v^{1}{)}_{j}=\sum_{j=1}^{i_{1}-1}(v^{1}{)}_{j}+(v^{1}{)}_{i_{1}}=\sum_{j=1}^{i_{1}-1}(v^{2}{)}_{j}+(v^{2}{)}_{i_{1}}+1=(\phi^{-1}(v^{2}){)}_{i_{1}}+1. \end{array}$$ (3.25)

Moreover, for the indices *i* such that $i_{1}<i<i_{2}$ (if any), we obtain


$$\begin{array}{cc} (\phi^{-1}(v^{1}){)}_{i} & =\sum_{j=1}^{i}(v^{1}{)}_{j}=\sum_{j=1}^{i_{1}}(v^{1}{)}_{j}+\sum_{j=i_{1}+1}^{i}(v^{1}{)}_{j}\\ & =\sum_{j=1}^{i_{1}}(v^{2}{)}_{j}+1+\sum_{j=i_{1}+1}^{i}(v^{2}{)}_{j}=(\phi^{-1}(v^{2}){)}_{i}+1. \end{array}$$ (3.26)

with


$$\begin{array}{c} (\phi^{-1}(v^{1}){)}_{i_{2}}=\sum_{j=1}^{i_{2}}(v^{1}{)}_{j}=\sum_{j=1}^{i_{2}-1}(v^{1}{)}_{j}+(v^{1}{)}_{i_{2}}=\sum_{j=1}^{i_{2}-1}(v^{2}{)}_{j}+1+(v^{2}{)}_{i_{2}}-1=(\phi^{-1}(v^{2}){)}_{i_{2}}. \end{array}$$ (3.27)

Using (3.24)–(3.27) and iterating the same argument with *i*_3_, *i*_4_, etc., we obtain that


$$\begin{array}{c} (\phi^{-1}(v^{1})-\phi^{-1}(v^{2}){)}_{i}\in \{0,1\},\;\text{for }i=1,...,k-1, \end{array}$$

and then by (3.18), $\{\phi^{-1}(v^{1}),\phi^{-1}(v^{2})\}\in E_{k}^{\prime \prime }$.

So $G_{k}^{\prime \prime }$ is isomorphic to $G_{k}$ via the map (3.6), as desired. □

**Corollary 3.15** *The regular triangulation* 𝒯 *of* $\Delta_{k,q}$ *can be described as*


$$\begin{array}{cc} 𝒯=\{\mathrm{Conv}(v^{1},...,v^{k})\;|\; & v^{1},...,v^{k}\in \Delta_{k,q}\cap {\mathbb{Z}}^{k},\\ & \{v^{i},v^{j}\}\in E_{k},\text{ for all }1\leq i<j\leq k\}. \end{array}$$ (3.28)

*Proof.* By Definition 3.5 and the definition of ϕ in (3.6),


$$\begin{array}{c} 𝒯=\{\phi (\sigma (w,\pi ))|\sigma (w,\pi )\text{ is a simplex of }T_{k}\}. \end{array}$$ (3.29)

By Corollary 3.12, we have $T_{k}=T_{k}^{\prime \prime }$, so the simplices of $T_{k}$ are precisely the convex hulls $\mathrm{Conv}(w^{1},...,w^{k})$, where $w^{1},...,w^{k}$ are pairwise adjacent in $G_{k}^{\prime \prime }$. Since ϕ defines a graph isomorphism between $G_{k}^{\prime \prime }$ and $G_{k}$ by Proposition 3.14, the image of each such simplex under ϕ is the convex hull of *k* vertices in $\Delta_{k,q}\cap {\mathbb{Z}}^{k}$ that are pairwise adjacent in $G_{k}$. Therefore, (3.29) is equivalent to (3.28), which proves the result. □

## 4. The lower bound

In order to establish a lower bound $\Omega (q^{k-2})$ for the number of non-monochromatic simplices, we need a preliminary definition of a hypergraph included in [6].

**Definition 4.1** The *Simplex-Lattice Hypergraph* $H_{k,q}$ is a pair $\left(V_{k,q},E_{k,q}\right)$, where*:*

- The vertex set is $V_{k,q}=\Delta_{k,q}\cap {\mathbb{Z}}^{k}$.
- The hyperedge set is
  $$\begin{array}{cc} E_{k,q}=\{\{ & (b_{1}+1,b_{2},...,b_{k}),\;(b_{1},b_{2}+1,...,b_{k}),\;...,\\ & (b_{1},b_{2},...,b_{k}+1)\}\;|\;(b_{1},...,b_{k})\in V_{k,q-1}\}. \end{array}$$ (4.1)

**Definition 4.2.** Let *T* be a triangulation of $\Delta_{k,q},$ whose vertices are labeled with a Sperner labeling c:V→{1,...,k}. A simplex in *T* is said to be monochromatic if all its vertices are assigned the same label by *c;* otherwise, the simplex is called *non-monochromatic.*

We denote by $m_{k,q}$ the *minimum number of non-monochromatic simplices* that appear in any Sperner labeling of the regular triangulation 𝒯 of $\Delta_{k,q}$, and we shall prove the bound given in (1.1).

*Proof.* The simplices associated with the hyperedges in (4.1) are simplices in the regular triangulation 𝒯. Indeed, observe that for any two vertices


$$\begin{array}{c} (b_{1},...,b_{i}+1,...,b_{j},...,b_{k})\;\text{and}\;(b_{1},...,b_{i},...,b_{j}+1,...,b_{k}), \end{array}$$ (4.2)

the difference is


$$\begin{array}{c} (0,...,0,-1,0,...,0,1,0,...,0), \end{array}$$ (4.3)

which satisfies (3.17). Then, all vertices in each hyperedge of $E_{k,q}$ form a simplex in 𝒯.

Therefore, the number of non-monochromatic simplices in 𝒯 is at least the number of non-monochromatic hyperedges in $E_{k,q}$. According to Proposition 2.1 of [6], this number is bounded below by (q+k−3k−2). □

Let us see an example for which the lower bound of Theorem 1.1 is not tight.

**Example 4.3** For *q =* 2, any Sperner labeling of the vertices of the regular triangulation 𝒯 has at most one monochromatic simplex.

Indeed, if every monochromatic simplex has label *k* (for instance), then the monochromatic simplices have the vertices either in $x_{k}=1$ or in $x_{k}=2$. But for *q =* 2, there is just one simplex with this property: the simplex with k−1 vertices in $x_{k}=1$ and a vertex in $x_{k}=2$, namely (0,...,0,2)*.*

If there exist monochromatic simplices with labels 1 and *k* (for instance), then we would have only one simplex with label 1 and only one simplex with label *k* as we have seen before, so the k−1 vertices in $V_{k}\cap \{x_{1}=1\}$ would have label 1 and the k−1 vertices in $V_{k}\cap \{x_{k}=1\}$ would have label *k,* so we obtain vertices in


$$\begin{array}{c} V_{k}\cap \{(x_{1}...,x_{k})|\;\;x_{1}=x_{k}=1\} \end{array}$$

with two labels, a contradiction.

Therefore, the number of non-monochromatic simplices is at least $2^{k-1}-1$, and then $m_{k,2}\geq 2^{k-1}-1$. This bound improves the lower bound (1.1), for *k >* 2.

## 5. An upper bound of $m_{k,q}$m(k,q)

We now establish an upper bound for $m_{k,q}$.

*Proof of Theorem 1.2* Consider the first choice labeling (Definition 2.4). In this labeling, a simplex is non-monochromatic if and only if it contains at least one vertex with first coordinate *x*_1_ = 0. These are all the simplices of the regular triangulation 𝒯 except for the simplices such that their vertices have first coordinate $x_{1}\geq 1$.

Since the simplices of the regular triangulation 𝒯 with a vertex in *x*_1_ = 0 are included in $x_{1}\leq 1$, then the simplices of 𝒯 such that their vertices have first coordinate $x_{1}\geq 1$ triangulate:


$$\begin{array}{c} \left\{(x_{1},...,x_{k})\in {\mathbb{R}}^{k}\;|\;x_{1}\geq 1,\;x_{2},...,x_{k}\geq 0,\;x_{1}+\cdots +x_{k}=q\right\}≅\Delta_{k,q-1}. \end{array}$$

so the number of said simplices is $(q-1{)}^{k-1}$ and then the number of non-monochromatic simplices for the first choice labeling is $q^{k-1}-(q-1{)}^{k-1}$. This implies that


$$\begin{array}{c} m_{k,q}\leq q^{k-1}-(q-1{)}^{k-1}, \end{array}$$

as desired. □

**Remark 5.1** By (1.2), we have


$$\begin{array}{c} m_{k,q}\leq q^{k-1}-(q-1{)}^{k-1}\sim (k-1)q^{k-2}. \end{array}$$

Hence, the multiplicative constant in the upper bound (1.2) tends to infinity as k→∞, whereas the multiplicative constant in the lower bound (1.1) tends to 0 as k→∞.

**Remark 5.2** For *k =* 2*, q =* 1, or *q = 2,* the lower bounds (1.1) and in Example 4.3 coincide with the upper bound (1.2). Therefore,


$$\begin{array}{c} m_{2,q}=m_{k,1}=1,\;m_{k,2}=2^{k-1}-1. \end{array}$$

## 6. Conclusions and future directions

In this paper, we address the open problem posed in [6] of determining the minimum number $m_{k,q}$ of non-monochromatic simplices in Sperner labelings of triangulations of $\Delta_{k,q}$. In that work, the authors emphasised the importance, for their applications, of obtaining a lower bound for $m_{k,q}$ with a tight multiplicative constant.

To this end, we characterise the regular triangulation 𝒯 of $\Delta_{k,q}$ and establish the bounds (1.1) and (1.2) for $m_{k,q}$, namely a lower bound of order $\Omega (q^{k-2})$ and an upper bound of order $O(q^{k-2})$. In addition, for the initial cases *k* = 2, *q* = 1, and *q* = 2, we determine the exact value of $m_{k,q}$. Therefore, for fixed *k*, our results determine the order of growth of $m_{k,q}$ for the regular triangulation and reduce the problem to finding the optimal multiplicative constant.

**Fig 1:**
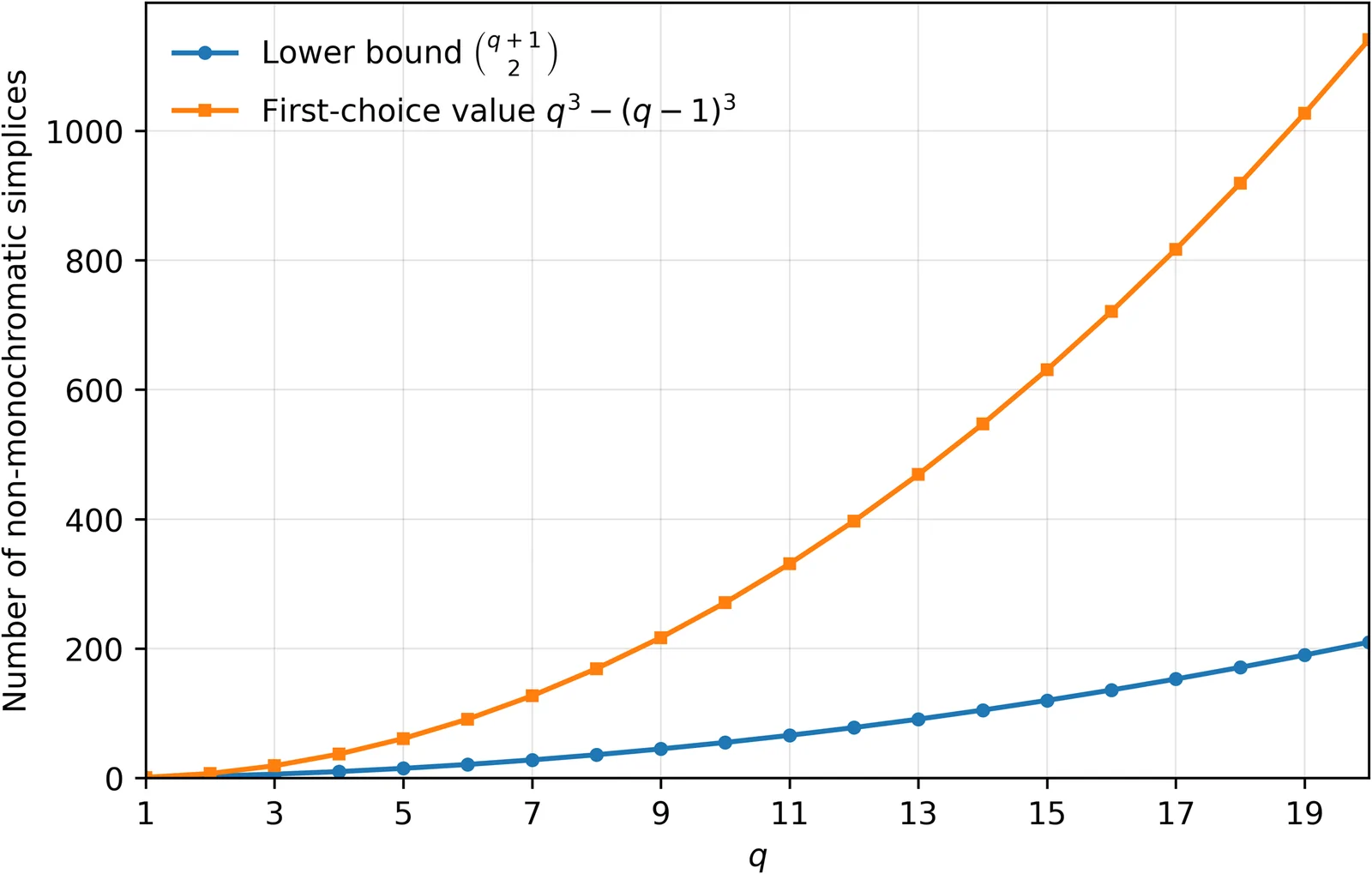
Comparison of lower bound and first choice $m_{k,q}$ for *k* = 4.

A natural direction for future work could be to narrow the gap between the multiplicative constant $\frac{1}{(k-2)!}$ of the lower bound of $m_{k,q}$ (Theorem 1.1) and the multiplicative constant k−1 in the upper bound (1.2). See Fig 1 for a visualisation of this gap.

In the initial cases for which we have established the exact value of $m_{k,q}$ (*k* = 2, *q* = 1, *q* = 2), this value is attained by the first choice labeling. We conjecture that this holds in general.

Our results suggest several research directions for fair division, hypergraph coloring, and combinatorial topology.

Determining the exact constant, or even the lower-order terms, in $m_{k,q}$ would sharpen worst-case guarantees for discrete fair-division algorithms. Classical rental-harmony models based on Sperner’s lemma [10], bounded envy-free cake-cutting protocols [2,11,12], and recent studies on mixed-resource division [13] all rely on understanding the structure of worst-case instances. Identifying the true minimum number of non-monochromatic simplices would help characterise these challenging cases and support the design of more robust allocation methods.

Refining the value of $m_{k,q}$ could impact rainbow generalisations of the KKM lemma and multi-cut problems such as necklace splitting and the Hobby-rice theorem, where multiple balanced partitions are sought simultaneously. Key developments in this direction include degree-theoretic and combinatorial proofs of Sperner-type results [14–17], polytopal generalisations [18], purely combinatorial approaches [19], rainbow and criticality extensions [20,21], and recent homotopy-based methods [22].

Finally, the regular triangulation considered here is closely related to the edgewise subdivision of a simplex [9] and to earlier work on simplicial mesh generation [8]. A more detailed geometric analysis of these constructions could lead to new algorithmic insights and improved combinatorial bounds.
